# Impact of Pulmonary microbiota on lung cancer treatment-related pneumonia

**DOI:** 10.7150/jca.93818

**Published:** 2024-06-17

**Authors:** Maoyuan Zhao, Wang Hou, Dan Pu, Zhixi Li, Li Tu, Calista Jia Ling Ow, Jie Tian, Weimin Li

**Affiliations:** 1 Lung cancer center, Frontiers Science Center for Disease-related Molecular Network, West China Hospital, Sichuan University, Chengdu, Sichuan, China.; 2 Department of Respiratory and Critical Care Medicine, Frontiers Science Center for Disease-related Molecular Network, West China Hospital, Sichuan University, Chengdu, Sichuan, China.; 3 Lung cancer center, West China Hospital, Sichuan University, Chengdu, Sichuan, China.; 4 BSc (Hons) Biochemistry, School of Biological Sciences, The University of Manchester, Manchester, United Kingdom.; 5 Department of Thoracic Surgery, West China Hospital, Sichuan University, Chengdu, Sichuan, China; 6 Precision Medicine Research Center, West China Hospital, Sichuan University, Chengdu, Sichuan, China.; 7 Research Units of West China, Chinese Academy of Medical Sciences, West China Hospital, Chengdu, Sichuan, China.

**Keywords:** lung cancer, pneumonia, microbiota, radiotherapy, PD-1

## Abstract

**Background:** The use of immunotherapy is progressively expanding for the treatment of lung cancer, either alone or in combination with radiotherapy. However, treatment-related adverse events, especially pneumonia, significantly limit the drug's effectiveness in treating lung cancer. The occurrence of lung cancer, immunotherapy, and pulmonary radiotherapy can all contribute to the imbalance in the pulmonary microbiota, rendering the lungs more susceptible to inflammatory reactions.

**Methods:** Mouse models of lung transplantation tumor were treated with either PD-1 monoclonal antibody or radiotherapy alone, or in combination. The differences in lung inflammation among the different treatment groups were regularly observed by micro-CT. Further, bronchoalveolar lavage fluid was extracted for macrogenomic and cytokine detection. The transcriptional genome of tumor-filled lung tissue was also sequenced.

**Results:** When treated with a combination of PD-1 and radiotherapy, the CT scans showed more severe pulmonary inflammation. However, with the addition of continuously administered antibiotics, no exacerbation of pneumonia signs was observed. Moreover, the differential gene expression and cytokine profiles in the combination treatment group differed from those in the PD-1 monotherapy group and the radiotherapy monotherapy group. This discrepancy does not seem to be a straightforward superimposition of radiation-induced pneumonia and immune-related pneumonia. Further exploration of changes in pulmonary microbiota revealed specific bacterial interactions with DEGs and cytokines.

**Conclusions:** The underlying causes of this susceptibility are intricate and may be associated with the complexity of pulmonary microbiota imbalance, along with fluctuations in the abundance of specific microbiota species.

## Introduction

Lung cancer remains a significant global health concern, and advancements in therapeutic strategies are continually sought to improve patient outcomes 1. In recent years, the combination of radiotherapy and immunotherapy has emerged as a promising approach, showing remarkable efficacy in various malignancies 2, including lung cancer 3. Combining radiotherapy with immunotherapy not only preserves the therapeutic efficacy of chemotherapy and immunotherapy but also increases the incidence of adverse events 4. Notably, the highest incidence was pulmonary adverse events exhibit the highest incidence 4. However, in this treatment approach, 67.6% of individuals experienced varying degrees of treatment-related pneumonia, with 11.8% of patients encountering Grade 3 or 4 pneumonia, necessitating the temporary suspension or interruption of treatment 4, 5.

The occurrence of adverse pulmonary reactions, mainly pneumonitis and pulmonary fibrosis, varies among individuals for reasons that have not been fully investigated scientifically. Over the past decades, the cGAS-STING pathway, which is closely associated with the recognition of microbial nucleic acids and the activation of innate immune responses, has been gradually elucidated. Currently, it is considered one of the most significant pathways involved in the combined immunotherapy of lung tumors 6, 7. Cyclic dinucleotides of bacterial origin can bind to STING, initiating the transcription of the IFN-beta gene 8. Numerous studies have indicated that the lower respiratory tract harbors a microbiome, forming a distinct pulmonary ecosystem 9. Clinical retrospective investigations and fundamental research have demonstrated that the pulmonary microbiota plays a significant role in the occurrence and progression of lung cancer, influencing the immune microenvironment within the lungs and impacting the response of pulmonary tumors to immunotherapy and radiotherapy 10-12. Both airway epithelial cells and alveolar macrophages express pattern recognition receptors, including Toll-like receptors, to detect pathogen-associated molecular patterns (PAMPs), triggering either proinflammatory or anti-inflammatory responses. 13-15. The perturbation of lung homeostasis initiates a sequence involving dysbiosis and inflammation. Specifically, mucus production is augmented by inflammation. This causes surrounding temperature to increase, which in turn attenuates oxygen tension, and additional nutrients are provided within an environment otherwise lacking in nutrients. Further inflammation is promoted as airways of the lungs are subjected to new PAMPs when intra-alveolar catecholamines and cytokines are produced, which fosters the proliferation of non-commensal bacterial species that have heightened immunogenicity 16. The occurrence of tumors, pulmonary radiotherapy, and immunotherapy precisely disrupt the homeostasis of the pulmonary microbiota, leading to a susceptibility to microbial dysbiosis in the lungs. Currently, there is a scarcity of reports that comprehensively consider these three factors. This study aims to conduct comprehensive transcriptome sequencing on lung tissues using mice harboring lung metastatic tumors, induced through tail vein injection. Additionally, metagenomic and cytokine analyses were performed on bronchoalveolar lavage fluid. The primary objective is to elucidate the associations among specific bacteria, differentially expressed genes, and cytokines within the context of these contributing factors.

## Methods

### Cell line and culture窗体顶端

We obtained the Lewis lung cancer (LLC) cells from the National Experimental Cell Resource Collection of the Chinese Academy of Medical Sciences/Peking Union Medical College (crcpumc, Beijing, China). The cultural environment was described elsewhere 17.

### The *in vivo* model

C57BL/6J mice (female, 4-week-old; Beijing HFK Bioscience Co., Ltd., Beijing, China) were housed in constant laboratory conditions with a 12-h light/dark cycle. 1-5x10^5^ LLC cells in 100 μl PBS were subcutaneously injected into the tail vein of the mice, with a total of 2 injections and a 3-day interval between each injection. One week before the intravenous injection of lung cancer cells, antibiotic treatment was initiated through the administration of antibiotics in the mice's drinking water. After 16 days, micro-CT images revealed the formation of lung tumors. Antibiotic administration was ceased in the non-antibiotic group. The specific experimental procedure is illustrated in Supplementary Figure 1. We randomly assigned those mice with pulmonary metastatic tumors shown in chest micro-CT scans into four groups: A) 100 μl normal saline; B) PD-1 (10 mg/kg/w); C) Radiotherapy (12Gy/F); D) antibiotic; E) PD-1+ Radiotherapy; F) PD-1 + antibiotic; G) Radiotherapy + antibiotic; H) PD-1 + Radiotherapy + antibiotic. Each group had 6 mice. The antibiotic drinking water comprised of metronidazole (1 g/L), neomycin (1 g/L), ampicillin (1 g/L), and vancomycin (0.5 g/L). PD-1 dissolved in normal saline was administered through intraperitoneal injection every two days for 12 days, where the first PD-1 treatment was given simultaneously with radiotherapy. The tumor burden of the lung with micro-computerized tomography (micro-CT, the eXplore Locus *in vivo* Micro-CT scanner, GE Healthcare) was monitored once a week. The first micro-CT scan was conducted the day before treatment, confirming the successful establishment of the model. The second micro-CT scan was performed on the 6th day of treatment to observe pulmonary inflammatory responses and tumor burden. The mice were euthanized 12 days after treatment. Subsequently, a small incision was made at the throat of the mouse, and a slender tube that was thinner than the mouse's trachea was inserted. The other end of the tube was connected to a 1 ml syringe containing normal saline, and the lungs of the mouse were lavaged to obtain bronchoalveolar lavage fluid. Each mouse was lavaged three times using a total of 1 ml of normal saline, yielding approximately 800 μl of bronchoalveolar lavage fluid from each mouse. The bronchoalveolar lavage fluid was then utilized for metagenomic sequencing and cytokine detection, while lung tissue was employed for RNA-seq sequencing. The breeding and handling of all mice comply with the relevant regulations of experimental animal ethics in China. The ethical record number for animal experiments is 20220217002.

### DNA isolation and metagenomics library preparation and sequencing

Total microbial genomic DNA samples were extracted using the Mag-Bind Soil DNA Kit (M5635-02) (OmegaBio-Tek, Norcross, GA, USA), following the manufacturer's instructions, and stored at -20°C prior to further assessment. The quantity and quality of extracted DNAs were measured using a NanoDrop ND-1000 spectrophotometer (Thermo Fisher Scientific, Waltham, MA, USA) and agarose gel electrophoresis, respectively. The extracted microbial DNA was processed to construct metagenome shotgun sequencing libraries with insert sizes of 400 bp by using the Illumina TruSeq Nano DNA LT Library Preparation Kit. Each library was sequenced by the Illumina HiSeq X-ten platform (Illumina, USA) with PE150 strategy at Personal Biotechnology Co., Ltd. (Shanghai, China). Raw sequencing reads were processed to obtain quality-filtered reads for further analysis. First, sequencing adapters were removed from sequencing reads using Cutadapt (v1.2.1). Secondly, low-quality reads were trimmed by using a sliding-window algorithm. Thirdly, reads were aligned to the host genome using BWA (http://bio-bwa.sourceforge.net/) 18 to remove host contamination. Once quality-filtered reads were obtained, they were de novo assembled to construct the metagenome for each sample by IDBA-UD (Iterative De Bruijn graph Assembler for sequencing data with highly Uneven Depth) 19. All coding regions (CDS) of metagenomic scaffolds longer than 300 bp were predicted by MetaGeneMark (http://exon.gatech.edu/GeneMark/metagenome) 20. CDS sequences of all samples were clustered by CD-HIT 21 at 90% protein sequence identity, to obtain a non-redundant gene catalog. Gene abundance in each sample was estimated by soap.coverage (http://soap.genomics.org.cn/) based on the number of aligned reads. The lowest common ancestor taxonomy of the non-redundant genes was obtained by aligning them against the NCBI-NT database by BLASTN (e value < 0.001). Similarly, the functional profiles of the non-redundant genes were obtained by annotating against the GO, KEGG, EggNOG, and CAZy databases, respectively, by using the DIAMOND 22 alignment algorithm. Based on the taxonomic and functional profiles of non-redundant genes, LEfSe (Linear discriminant analysis effect size) was performed to detect differentially abundant taxa and functions across groups using the default parameters 23. Beta diversity analysis was performed to investigate the compositional and functional variation of microbial communities across samples using Bray-Curtis distance metrics and visualized via principal coordinate analysis (PCoA), nonmetric multidimensional scaling (NMDS), and unweighted pair-group method with arithmetic means (UPGMA) hierarchical clustering 24.

### RNA sequencing

Total RNA was isolated using the Trizol Reagent (Invitrogen Life Technologies), after which the concentration, quality, and integrity were determined using a NanoDrop spectrophotometer (Thermo Scientific). Three micrograms of RNA were used as input material for the RNA sample preparations. Sequencing libraries were generated using the TruSeq RNA Sample Preparation Kit (Illumina, San Diego, CA, USA). Briefly, mRNA was purified from total RNA using poly-T oligo-attached magnetic beads. Fragmentation was carried out using divalent cations under elevated temperature in an Illumina proprietary fragmentation buffer. First strand cDNA was synthesized using random oligonucleotides and SuperScript II. Second strand cDNA synthesis was subsequently performed using DNA Polymerase I and RNase H. Remaining overhangs were converted into blunt ends via exonuclease/polymerase activities and the enzymes were removed. After adenylation of the 3′ ends of the DNA fragments, Illumina PE adapter oligonucleotides were ligated to prepare for hybridization. To select cDNA fragments of the preferred 200 bp in length, the library fragments were purified using the AMPure XP system (Beckman Coulter, Beverly, CA, USA). DNA fragments with ligated adaptor molecules on both ends were selectively enriched using Illumina PCR Primer Cocktail in a 15 cycle PCR reaction. Products were purified (AMPure XP system) and quantified using the Agilent high sensitivity DNA assay on a Bioanalyzer 2100 system (Agilent). The sequencing library was then sequenced on a Hiseq platform (Illumina) by Shanghai Personal Biotechnology Cp. Ltd.

### DEGs screening, PPI network construction

Differential expression analysis between two comparison groups was conducted using DESeq software (version1.20.0). DESeq was employed for differential analysis of gene expression, with the criteria for selecting differentially expressed genes set as expression fold change |log2FoldChange| > 1, and significance level P-value < 0.05. A web tool, the STRING database (https://string-db.org/), was used to build a protein-protein interaction network for the DEGs 25, and the minimum required interaction score was set as 0.700. The MCODE plugin was utilized to identify subnetworks.

### Enrichment analysis of differential genes

We performed Gene Ontology (GO) enrichment analysis using top GO. The P-value was calculated through the hypergeometric distribution method, with a significance threshold set at P-value < 0.05. This allowed us to identify significantly enriched GO terms associated with differentially expressed genes, elucidating the main biological functions influenced by these genes. Additionally, we employed clusterProfiler (version 3.4.4) software for the Kyoto Encyclopedia of Genes and Genomes (KEGG) pathway enrichment analysis, focusing on pathways with a significant enrichment indicated by a P-value < 0.05.

### Cytokine detection

The protein concentration is determined by the BCA Protein Assay Kit (PA115, TIAN GEN, Beijing). Protein array membranes were blocked in blocking buffer for 30 minutes and then incubated with samples at room temperature for 1 to 2h (or incubated at 40C overnight). After decanting the samples, membranes were washed with washing buffer and then incubated with diluted biotin-conjugated antibodies at room temperature for 1-2 h. The membranes were washed again before adding streptavidin-conjugated fluor at room temperature. Membranes were given a final wash prior to being scanned by the Axon scanner. By comparing the signal intensities, relative expression levels of cytokines were made. The intensities of signals are quantified by densitometry. Raw intensities were revised by background and normalized by median. Fold changes in protein expression were calculated.

### Network analysis of dominant species

The Mothur software was used to compute Spearman rank correlation coefficients among dominant species. A correlation network was constructed for those species exhibiting |rho| > 0.6 and a P-value < 0.01.

## Results

### Observation of pulmonary inflammatory response by micro-CT scanning

**Figure 1:**
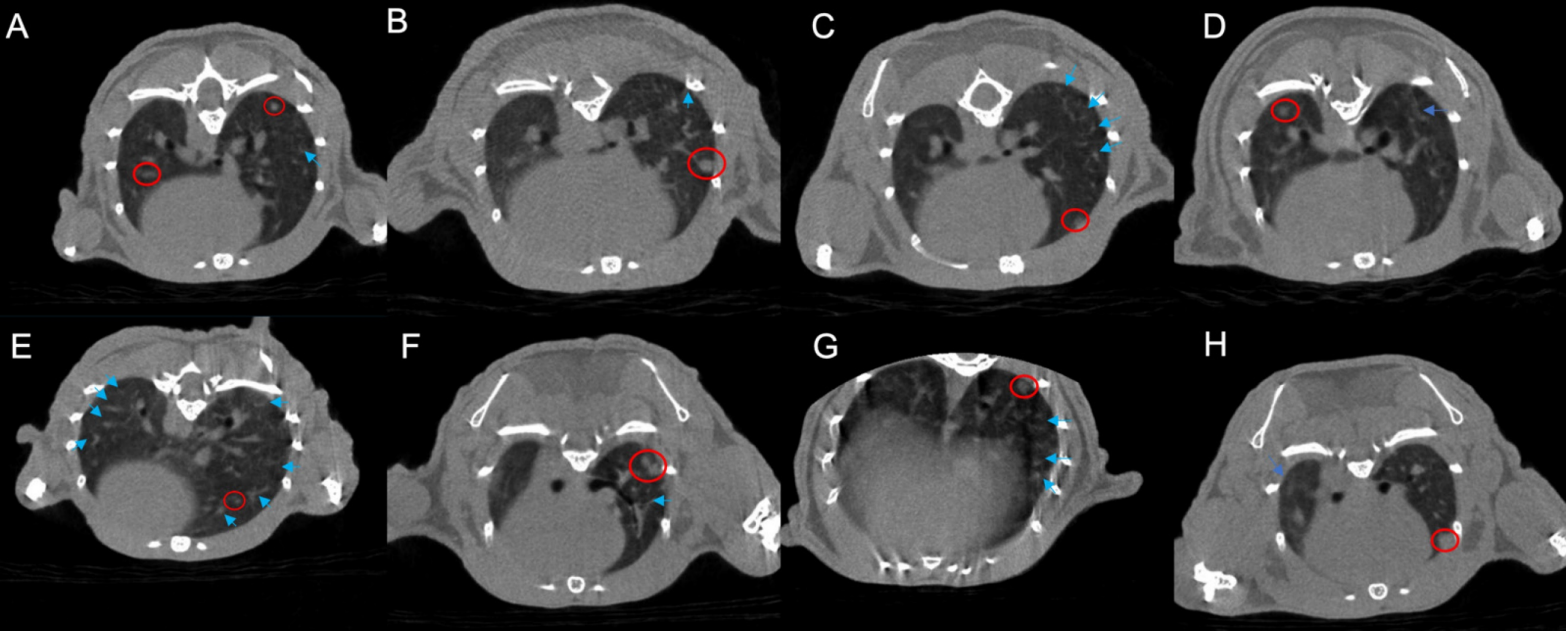
Micro-CT images of lung metastases in tumor-bearing mice across the eight experimental groups. A. control; B. PD-1; C. Radiotherapy; D. antibiotic; E. PD-1 + Radiotherapy; F. PD-1 + antibiotic; G. Radiotherapy + antibiotic; H. PD-1 + Radiotherapy + antibiotic. The bright blue arrows point to areas of inflammation in the lungs, and the bright red circle shows the tumor.

Based on CT images, the radiotherapy group exhibits a greater manifestation of pulmonary inflammation compared to the PD-1 group. This observation is evident in both the comparison between the monotherapy radiotherapy group and the PD-1 group, as well as in the comparison between the combination therapy of radiotherapy and antibiotics and the combination therapy of PD-1 and antibiotics. Furthermore, our primary focus, the group that received PD-1 combined with radiotherapy, indeed demonstrates the highest degree of pulmonary inflammation among the eight groups. Interestingly, the triple combination group of PD-1/radiotherapy/antibiotics does not exhibit a higher level of pulmonary inflammation than the radiotherapy monotherapy group (Figure 1).

### RNA sequencing showed DEGs

**Figure 2:**
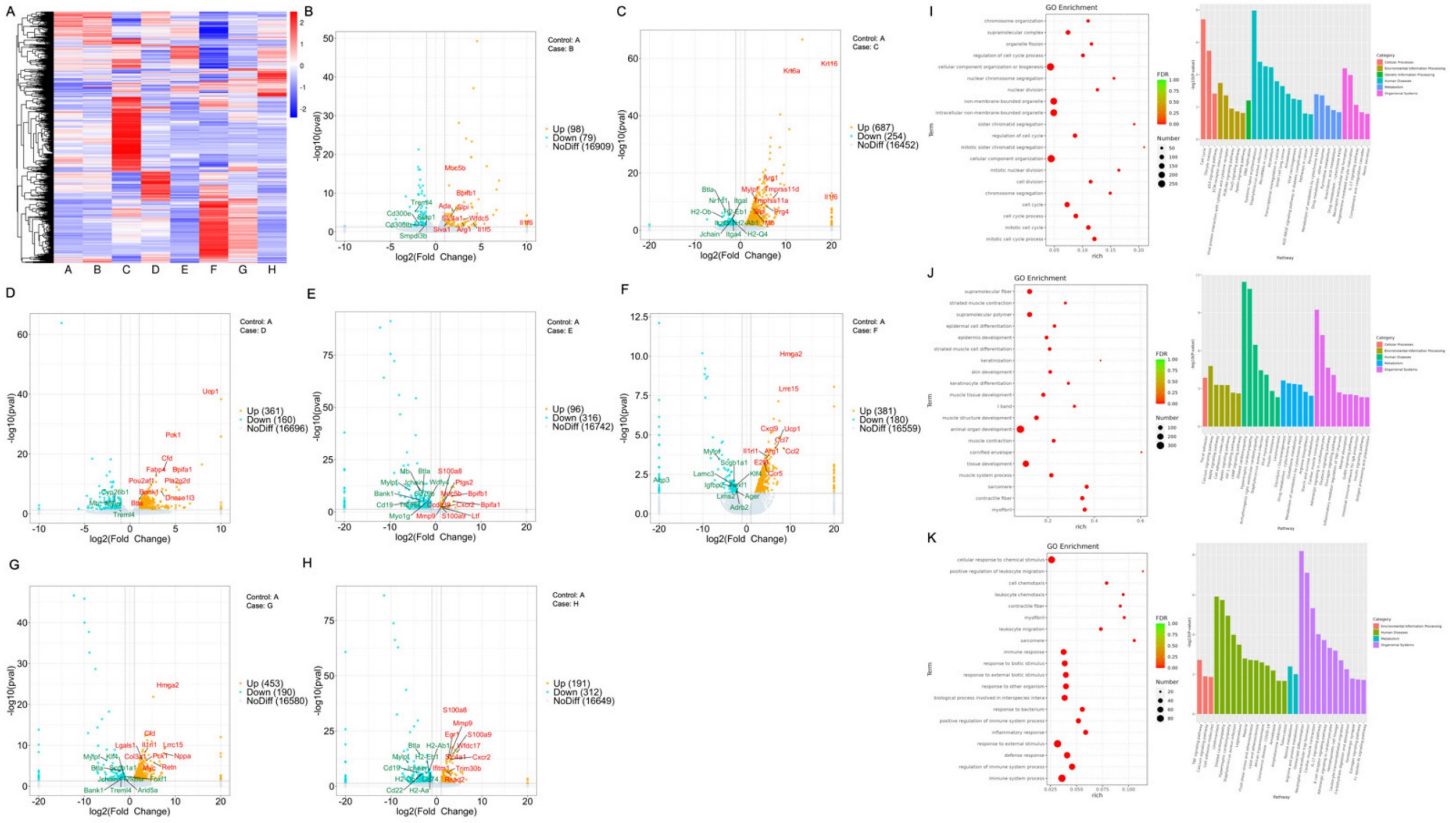
DEGs analysis among different treatment groups. A. DEGs are shown in the heatmap. B-I: Displaying a volcano plot for DEGs in each group: the horizontal axis represents log2FoldChange, and the vertical axis represents -log10(p-value). Two vertical dashed lines in the plot indicate a 2-fold expression difference threshold, while the horizontal dashed line represents the P-value=0.05 threshold. Orange dots represent upregulated genes in the group, blue dots represent downregulated genes in the group. The genes relevant for subsequent screening are annotated. C. A vs B; D. A vs C; E. A vs D; F. A vs E; G. A vs F; H. A vs G; I. A vs H. A. The GO and KEGG of DEGs, group PD-1 vs the combination (I), group radiotherapy vs the combination (J), group PD-1 with radiotherapy vs the group PD-1 with radiotherapy plus antibiotic (K). The specific grouping information for the mice was provided in the methods.

RNA sequencing was conducted on the lung tissues of the mouse, while the bronchoalveolar lavage fluid was used for cytokine detection to investigate the inherent mechanism more thoroughly. Precisely, changes related to immunity and inflammation were observed. Although different levels of pneumonia severity were observed in the eight groups based on previous micro-CT scans, the expressions of genes showed pronounced variations (Figure 2A). Differential genes for each group are visually represented through volcano plots (Figure 2B-H). Further, we performed cluster analysis on DEGs. The findings were elucidated using GO and KEGG pathway annotations. When comparing between the group receiving both PD-1 and antibiotics with the group undergoing PD-1 alone, the top three enriched cellular components (CC) were intracellular non membrane bounded organelle, non-membrane bounded organelle, and supramolecular complex. On the other hand, the main molecular functions (MF) were structural molecule activity, cytoskeletal protein binding, and binding, while the major biological processes (BP) revolved around the cell cycle. Simultaneously, these findings were associated with pathways related to the cell cycle, neutrophil activity, and certain pathways implicated in tumor alterations (Figure 2I). When this comparison was made within the radiotherapy group, we observed that the top three CCs were myofibril, contractile fiber, and cornified envelope. The core MFs were associated with protein binding, and the top three BP were related to muscle or tissue processes. KEGG pathways were enriched more in the MAPK signal pathway and Calcium pathway. Enrichment analysis in the KEGG also revealed enrichment in pathways such as IL-17 and TNF signaling pathway, among others (Figure 2J). When focusing on the combined radiotherapy and PD-1 group, notable patterns emerged in BP, indicating robust immune system processes. Moreover, the MF predominantly involved protein binding and other molecular functionalities aligning with active immune processes. Enrichment analysis in the KEGG also revealed enrichment in pathways such as IL-17 and TNF signaling, among others (Figure 2K).

### The pulmonary microbiotas vary across different treatment groups

**Figure 3:**
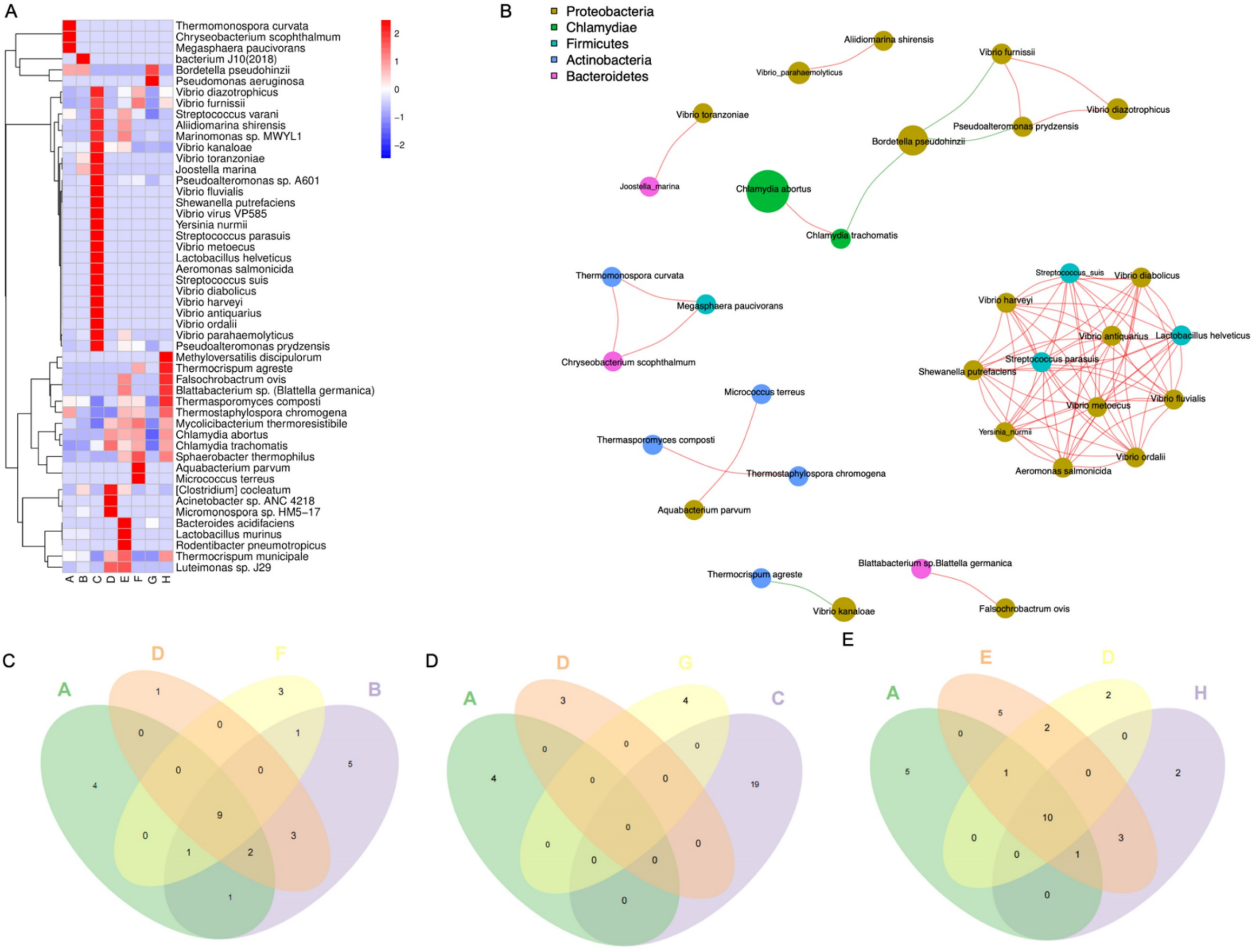
Pulmonary microbiotas vary across different treatment groups. A. Heat map illustrating the differences in microbial communities among different groups. B. Interactions among various microbiotas that are from five phylums, the red line represents a positive correlation, while the green line represents a negative correlation. C-E. The Venn diagram illustrating different microbiotas with or without the addition of antibiotics. The specific grouping (A-H) information for the mice was provided in the methods.

To further explore the impact of pulmonary microbiota on pneumonia, we observed and compared the pulmonary microbiotas within each experimental group under conditions of antibiotic administration or absence thereof. Metagenomic sequencing revealed the top 50 differentially abundant microbial species across the eight groups (Figure 3A). Some microbiotas were excluded from the analysis due to potential experimental contaminants, such as microbiotas associated with procedural interference or intestinal colonization. In the microbial community correlation network, Proteobacteria dominated the majority, with *Bordetella Pseudohinzii* exhibiting the highest abundance within this phylum. *Chlamydia abortus* exhibited the highest abundance, while *Chlamydia trachomatis*, belonging to the same phylum Chlamydiae, showed a negative correlation with the most abundant bacterium, *Bordetella pseudohinzii*, in the *Proteobacteria phylum*. Notably, there appeared to be a close relationship among members labeled as *Shewanella putrefaciens*, *Vibrio harveyi*, *Vibrio diabolicus*, *Vibrioantiquarius*, *Vibrio metoecus*, *Vibrio fluvialis*, *Yersinia_nurmii*, *Vibrioordalii*, *Aeromonas salmonicida*, *Streptococcus_suis*, *Lactobacillus helveticus*, *Streptococcus parasuis* (Figure 3B). The number and the name of the species of differentially abundant microbiotas that overlapped when PD-1 was combined with antibiotics (Figure 3C, Supplementary File 1A), when radiotherapy is combined with antibiotics (Figure 3D, Supplementary File 1B), and when radiotherapy was combined with PD-1 treatment (Figure 3E, Supplementary File 1C) are illustrated as Venn diagrams and table.

### Interactions among pulmonary microbiota, genes, and cytokines

**Figure 4:**
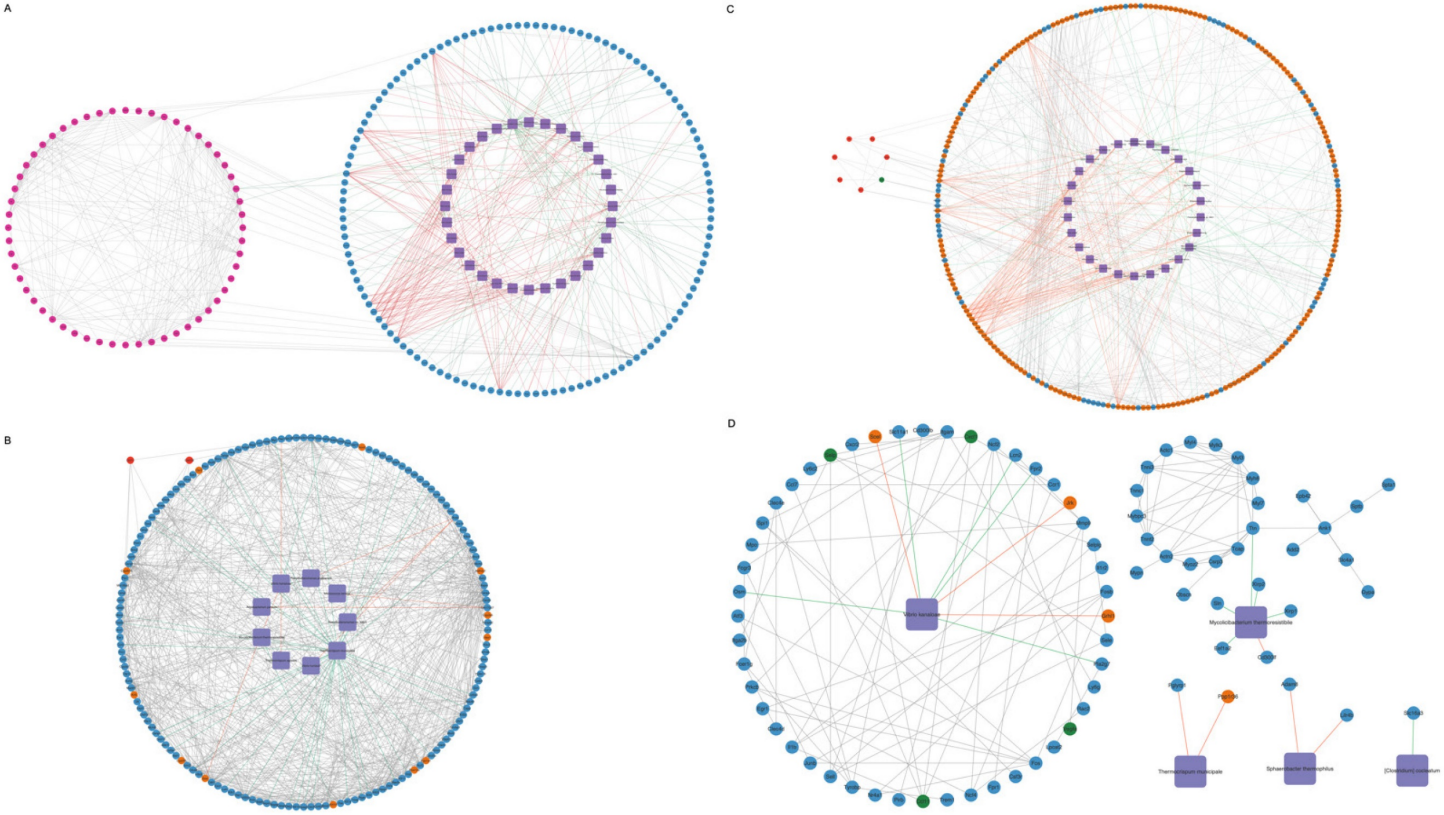
** Interactions among pulmonary microbiota, genes, and cytokines.** Purple rhombus represents microbial species; upregulated and downregulated cytokines are shown in red and green respectively; genes that are upregulated are in orange-yellow, while those that are downregulated are shown in blue. Interactions among microbial species, genes, and cytokines. Information of specific grouping (A-H) of the mice was provided in the methods. **(A)** The network of all gene-microbe-cytokine Spearman correlations. The gene-microbe-cytokine Spearman correlations network when group F is compared with group B **(B)**, group G with group C** (D)**, and lastly group H with group E **(D)**.

To explore the interactions among differential genes, cytokines, and pulmonary microbiota, a Spearman correlation analysis was conducted. This involved combining the 50 microbial species with the total set of differential genes, representing the union of differential genes in each comparison group, in conjunction with the overall cytokines. The resulting correlations encompass gene-microbe-cytokine Spearman correlations. Subsequently, refine the results by excluding genes and cytokines that do not exhibit direct or concise associations with the microbial species. Filtering relationships with correlation coefficients > 0.9 or <-0.9 and p < 0.05, construct the overall network, we generated a relationship network composed of 295 nodes and 666 edges, involving 32 microbial species, 56 cytokines, and 207 genes in the context of microbe-differential gene-cytokine interactions (Figure 4A, Supplementary File 2A). Between the PD-1 monotherapy groups with and without antibiotic usage, a correlation network composed of 167 nodes and 674 edges was constructed and 9 microbial species that significantly impact the lung microbiota were identified. These 9 microbial modulated 156 DEGs, where 144 genes were downregulated while 12 genes were shown to be upregulated. Additionally, this interaction resulted in the augmentation of the cytokines Ccl11 and Cxcl16 (Figure 4B, Supplementary File 2B). Next, when the radiotherapy monotherapy groups with and without antibiotic usage were compared, an interaction network with 284 nodes and 594 edges was obtained. Within this network, 26 microbial species interacted with 251 DEGs (189 upregulated and 62 downregulated), causing the upregulation of 6 cytokines (IL1a, Pf4, Ccl11, Cx3cl1, Xcl1, IL6) and the downregulation of cytokine Ccl5 (Figure 4C, Supplementary File 2C). Finally, when assessing the cohort receiving radiotherapy, both with and without antibiotics, in conjunction with PD-1, an interaction network with 87 nodes and 125 edges was revealed. Specifically, 5 microbial species namely *Vibrio Kanaloae*, *Mycolicibacterium thermoresistibile*, *Thermocrispum municipale*, *Sphaerobacter thermophilus*, and *[Clostridium] cocleatum*. were associated with 78 DEGs, in which most of the genes were diminished (n= 74) while only 4 showed elevated expression. Cytokines Ccl11, Vegfa, Selp, and Cxcl1 were also attenuated (Figure 4D, Supplementary File 2D).

## Discussion

Throughout the 20th century, the lung was considered a sterile environment highlighting the limitations of culture-based methods. However, with the implementation of culture-independent techniques in the 21st century, this dogma has been challenged 16. Previous studies have indicated the presence of microbial species in the human distal airways 26. In a state of health and symbiosis, the lung microbiome maintains a dynamic equilibrium, with symbiotic bacteria, airway epithelium, and alveolar macrophages in a tolerant state. When the lung loses its homeostasis, a dysbiosis-inflammatory cycle ensues 16. In diseased lungs, the delicate balance between microbial influx and clearance is disrupted, leading to dysbiosis, a phenomenon confirmed in various pulmonary diseases 27. The occurrence of lung cancer, immunotherapy disrupting immune checkpoint restrictions, and tumor radiotherapy simultaneously disrupting the DNA of tumor cells and local microbes all contribute to the disruption of lung microbial equilibrium 28-30. The use of antibiotics can alter the diversity of the gut microbiome, which may in turn affect the efficacy of immunotherapy. A study found that for patients with advanced renal cell carcinoma and non-small cell lung cancer, the use of antibiotics within the first 30 days of PD-1/PD-L1 antibody treatment was associated with a decreased survival rate and significantly shorter overall survival 31. There are numerous reports on the research of PD-L1/PD-1 immunotherapy and antibiotics, but the main focus is on the study of gut microbiota 32, 33, with less attention given to the lung microbiota. However, there is a significant difference between the gut microbiome and the lung microbiome. Previous researchers in our team have found that the microbiota of the lungs in lung cancer patients differs from that of healthy lungs 34.

Our study, building upon previous research, focused on the alterations in lung inflammation and pulmonary microbiota in a mouse model of lung metastasis following radiotherapy and/or immunotherapy. Our results elucidated the interactions between the mouse lower respiratory tract microbiome, differential genes in lung tissues, and cytokines in bronchoalveolar lavage fluid. The microbiota detected in this study are primarily concentrated in five phyla: Proteobacteria, Chlamydiae, Firmicutes, Actinobacteria, and Bacteroidetes. Regarding the relationship between Proteobacteria and tumors, studies have found that the gut microbiota may play a key role in the occurrence, progression, treatment, and prognosis of many types of cancer 35. The gut and lung microbiota could directly exchange microbial species through lymphatic circulation, and the bioactive metabolites produced by the gut microbiota and lung microbiota can change through blood circulation 36. The complex interactions between the lung microbiota, respiratory viruses, and the host immune system could regulate immune homeostasis and affect the inflammatory response in the lungs 37. Regarding the relationship between Proteobacteria and pneumonia, the upper respiratory tract and gut microbiota of healthy individuals protect the lungs from pneumonia by preventing the colonization of potential pathogens and regulating immune responses 38. Chlamydiae is a phylum of bacteria, among which *Chlamydia pneumoniae* is a common pathogen for respiratory infections, including pneumonia 39. Whether *Chlamydia pneumoniae* infection was an independent risk factor for lung cancer remains to be further studied 40. *Chlamydia pneumoniae* is a common cause of community-acquired pneumonia. However, not everyone exposed to *Chlamydia pneumoniae* developed pneumonia 41. Human microbiota, including Firmicutes, Actinobacteria and Bacteroidetes, might influence the occurrence of lung cancer through metabolic, inflammatory, or immune pathways, and also affect the efficacy of radiotherapy, gene therapy, and immunotherapy 30. There was almost no literature reporting a strong correlation between Firmicutes, Actinobacteria, and Bacteroidetes with acute or chronic inflammation in the lungs. Ultimately, we identified five prominent microbial species (*Vibrio Kanaloae*, *Mycolicibacterium thermoresistibile*, *Thermocrispum municipale*, *Sphaerobacter thermophilus*, and *[Clostridium] cocleatum*) and their interactive relationships with immune-related genes and cytokines. These microbiotas, genes, and cytokines might collectively play a role in controlling both tumors and treatment-related pulmonary inflammation.

While there is limited documentation on *Vibrio kanaloae*, a bacterium commonly associated with pathogenic effects on aquatic organisms, reports on its involvement in pulmonary diseases in humans or mice are scarce. However, other bacteria, within the Proteobacteria to which it belongs have shown significant associations with chronic inflammation in the lungs 36, 42. Besides, research suggests that gut microbiota, including Proteobacteria, can influence cancer development and therapeutic responses 43. It has been found that Proteobacteria are associated with poor treatment responses 43, in cancerous tissues, the presence of Proteobacteria is increased 44. It's also worth noting that the proliferation of Proteobacteria is associated with an increase in IL-6 and IL-8, which are inflammatory cytokines 45. This suggested a possible link between Proteobacteria and inflammation. *Mycolicibacterium brumae*, a non-tuberculous mycobacterium, has shown promising anti-tumor and immunomodulatory capabilities. It has been explored as an alternative therapy to Bacillus Calmette-Guérin (BCG) immunotherapy, which is the gold-standard treatment for non-muscle-invasive bladder cancer patients 46. Mycolicibacterium is often considered a commensal rather than a pathogenic bacterium. It is infrequently pathogenic, but not categorically benign 47. 1 described a rare case of pleural effusion caused by *Mycolicibacterium mageritense* in an immunocompetent patient 48. The patient had no history of immunodeficiency and no recurrence of lung cancer after surgery. However, 8 months after surgery, he developed a new lung shadow and pleurisy. This case emphasizes that nontuberculous mycobacterial pleurisy should be considered in the differential diagnoses of pleural effusion even in immunocompetent patients. *Thermocrispum municipale* and *Mycolicibacterium thermoresistibile* belong to the Actinobacteria phylum, but there is limited existing literature on the immunological aspects of *Thermocrispum municipale*. However, certain studies have underscored the enzymatic potential of *Thermocrispum municipale*, particularly cyclohexanone monooxygenase. This enzyme exhibited high efficiency in converting various aliphatic, aromatic, and cyclic ketones, as well as prochiral sulfides 49. In a study on lignans, a type of polyphenolic compound, it was mentioned that certain bacteria, including *Clostridium cocleatum*, could catalyze reactions involving these compounds2. The study also mentioned that in mouse models of oxidative lung injury, flaxseed, which is a rich source of lignans, has been shown to be radio-protective 50. Pre-treatment with a lignan called secoisolariciresinol diglucoside (SDG) was found to protect lung cells from radiation 50. The interaction between pulmonary microbiota and inflammation related to lung cancer treatment still requires further exploration. The revealed interactions between pulmonary microbiota and differential genes, as well as cytokines in this study, may offer some guidance for clinicians regarding treatment-related side effects. However, further validation through additional clinical testing is necessary in the future.

## Supplementary Material

Supplementary figures and data.

## Figures and Tables

**Table 1:** Differentially abundant microbiota that are overlapping when PD-1 was combined with antibiotics

Group	A	B	D	F
Microbiota phylum	*Chryseobacterium scophthalmum*	*Vibrio parahaemolyticus*	*Acinetobacter* sp. ANC 4218	*Aquabacterium parvum*
	*Megasphaera paucivorans*	*Vibrio toranzoniae*		*Micrococcus terreus*
	*Coprinopsis cinerea*	*bacterium* J10(2018)		*Thermocrispum agreste*
	*Thermomonospora curvata*	*Psychrobacter* sp. JB385		
		*Joostella marina*		

**Table 2:** Differentially abundant microbiota that are overlapping when radiotherapy was combined with antibiotics

Group	A	D	G	C
Microbiota phylum	*Chryseobacterium scophthalmum* *Megasphaera paucivorans* *Coprinopsis cinerea* *Thermomonospora curvata*	*Acinetobacter* sp. ANC 4218 *Micromonospora* sp. HM5-17 *Luteimonas* sp. J29	*Pseudomonas aeruginosa* *Bacteroides acidifaciens* *Thermocrispum agreste* *Sphaerobacter thermophilus*	*Vibrio parahaemolyticus* *Vibrio antiquaries* *Vibrio ordalii* *Vibrio toranzoniae* *Vibrio harveyi* *Paracoccus sediminis* *Vibrio diabolicus* *Streptococcus suis* *Aeromonas salmonicida* *Lactobacillus helveticus* *Vibrio metoecus* *Streptococcus parasuis* *Yersinia nurmii* *Vibrio virus* VP585 *Aliidiomarina shirensis* *Shewanella putrefaciens* *Vibrio fluvialis* *Joostella marina* *Psychrobacter* sp. JB385

**Table 3:** Microbiotas that differed yet overlapped when combining PD-1 and radiotherapy with antibiotics

Group	A	D	E	H
Microbiota phylum	*Chryseobacterium scophthalmum* *Megasphaera paucivorans* *Coprinopsis cinerea* *Thermomonospora curvata* *Bordetella pseudohinzii*	*Acinetobacter* sp. ANC 4218 *Micromonospora* sp. HM5-17	*Vibrio parahaemolyticus* *Rodentibacter pneumotropicus* *Bacteroides acidifaciens* *Aliidiomarina shirensis* *Marinomonas* sp. MWYL1	*Methyloversatilis discipulorum* *Thermocrispum agreste*
