# Supplementary material for: Impact of Pulmonary microbiota on lung cancer treatment-related pneumonia
Source: J Cancer. 2024 Jun 17;15(14):4503–12. doi: 10.7150/jca.93818 (PMC11242340; doi:10.7150/jca.93818)
Supplement: Supplementary file 1 — Supplementary figures and data. [file jcav15p4503s1.zip › s1/supplement files/supplementary file legends.pdf]

**Supplementary Figure 1**

Establishment of Mouse Pulmonary Metastasis Cancer Model and Treatment Timeline.

**Supplementary File 1A**

This file displays the differences and intersections of microbiota in the four sample groups A, B,D, and F.

**Supplementary File 1B**

This file displays the differences and intersections of microbiota in the four sample groups A, C,D, and G.

**Supplementary File 1C**

This file displays the differences and intersections of microbiota in the four sample groups A, D,E and H.

**Supplementary File 2A**

This file displays the gene names, microbiota names, and cytokine names in this network of all gene-microbe-cytokine Spearman correlations.

**Supplementary File 2B**

This file displays all the gene names, microbiota names, and cytokine names in this network of gene-microbe-cytokine Spearman correlations when group F is compared with group B.

**Supplementary File 2C**

This file displays all the gene names, microbiota names, and cytokine names in this network of gene-microbe-cytokine Spearman correlations when group G is compared with group C.

**Supplementary File 2D**

This file displays all the gene names, microbiota names, and cytokine names in this network of gene-microbe-cytokine Spearman correlations when group H is compared with group E.
