# Supplementary figures and images for: Impact of Pulmonary microbiota on lung cancer treatment-related pneumonia
Source: J Cancer. 2024 Jun 17;15(14):4503–12. doi: 10.7150/jca.93818 (PMC11242340; doi:10.7150/jca.93818)

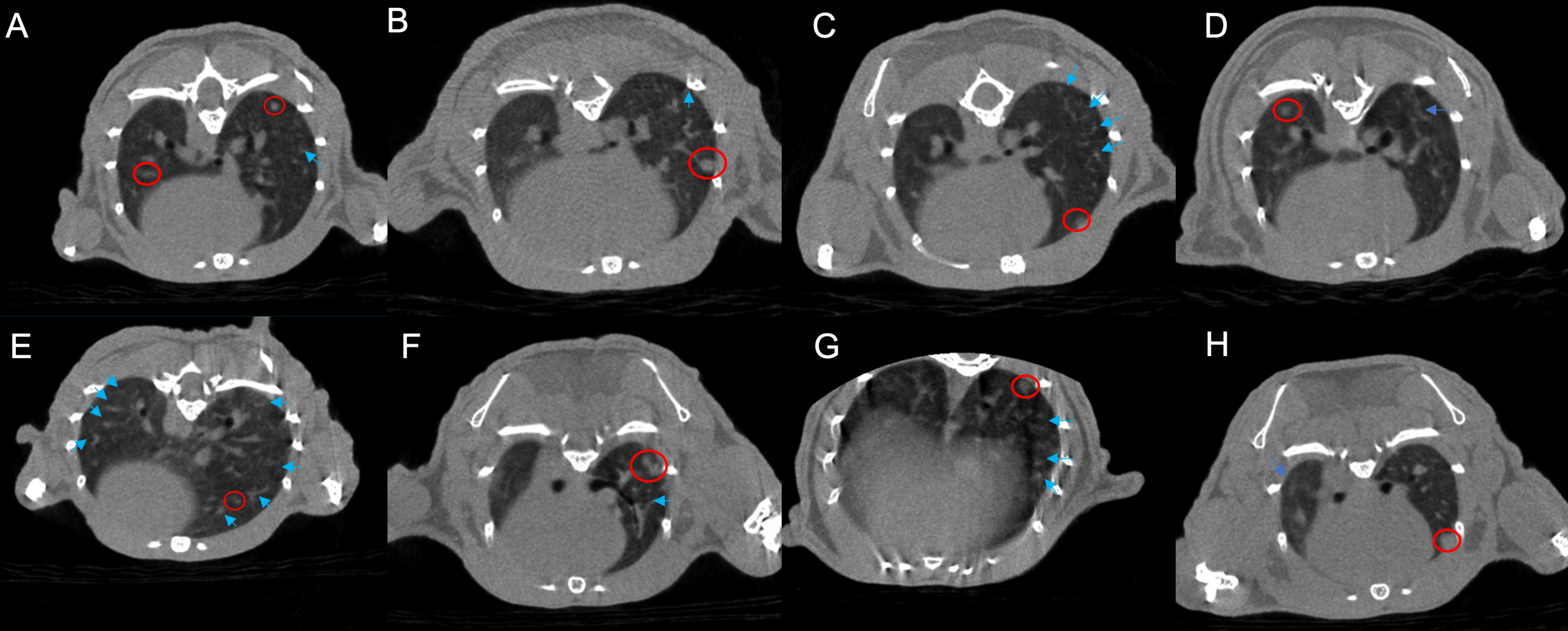

Supplement: Supplementary file 1 — Supplementary figures and data. [file jcav15p4503s1.zip › s1/supplement files/Figure 1R.tif]

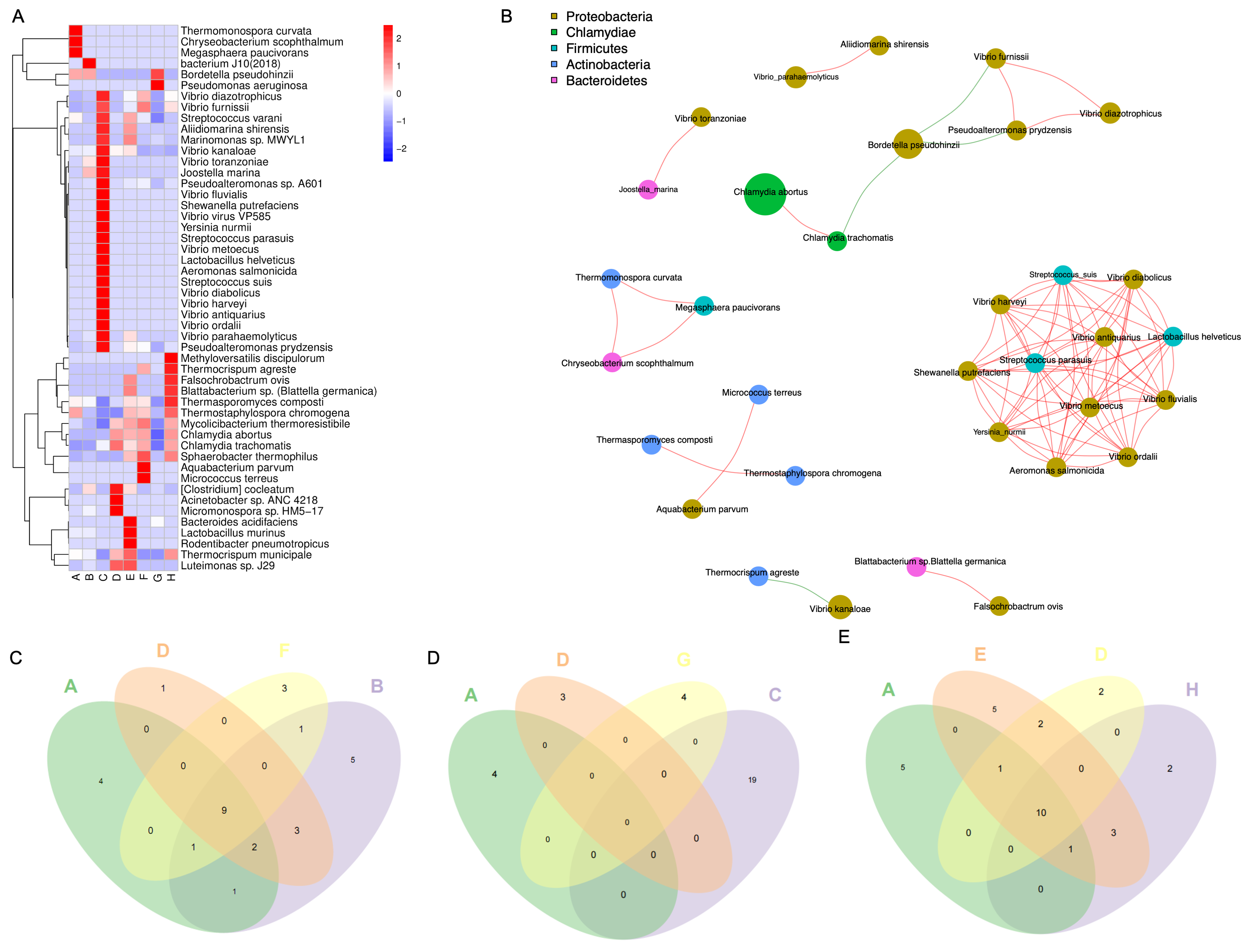

Supplement: Supplementary file 1 — Supplementary figures and data. [file jcav15p4503s1.zip › s1/supplement files/Figure 3.tiff]

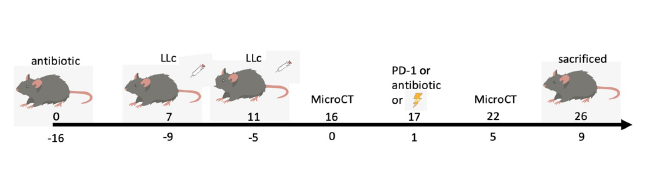

Supplement: Supplementary file 1 — Supplementary figures and data. [file jcav15p4503s1.zip › s1/supplement files/supplementary figure 1.tif]
